# Deep Postanal Abscess With Sacrococcygeal Osteomyelitis: A Case Report

**DOI:** 10.7759/cureus.80341

**Published:** 2025-03-10

**Authors:** Javid Ahmadov, Mustafa Anil Turhan, Ender Erguder, Sezai Leventoğlu, Bulent Mentes

**Affiliations:** 1 General Surgery and Proctology, Ankara Memorial Hospital, Ankara, TUR; 2 Transplant, Ankara Memorial Hospital, Ankara, TUR; 3 General Surgery, Ankara Etlik City Hospital, Ankara, TUR; 4 General Surgery, Gazi University Hospital, School of Medicine, Ankara, TUR; 5 General and Colorectal Surgery, Gazi University, Ankara, TUR

**Keywords:** coloproctology, deep pelvic abscess, draining seton, osteomyelitis, surgical techniques

## Abstract

Deep postanal abscesses (DPAs) are uncommon but clinically significant conditions that, if left untreated, can result in severe septic complications. This case report presents a rare instance of sacrococcygeal osteomyelitis as a complication of a long-standing DPA. The patient underwent surgical drainage and seton placement, leading to complete resolution of both the abscess and the osteomyelitis. This case underscores the importance of prompt surgical intervention in the management of DPAs and highlights the potential for rare but serious complications such as osteomyelitis.

## Introduction

Deep postanal abscesses (DPAs) are complex conditions that challenge clinicians due to their location, diverse symptomatology, and difficulties in surgical management. The deep postanal space is defined as the area bounded anteriorly by the external anal sphincter (EAS) complex, posteriorly and inferiorly by the coccyx and anococcygeal ligament, and superiorly by the levator muscle [1]. Deep posterior anal fistulas typically result from infections of the anal glands (referred to as the internal opening or primary orifice) due to glandular obstruction [2]. An infected anal gland forms an abscess in the posterior intersphincteric region, which can extend beyond the EAS into the ischiorectal fossa. This extension results in external openings (secondary orifices) in the perianal skin, which can be unilateral or bilateral [3]. These infected glands originating from the posterior midline can traverse the conjoint longitudinal muscle and reach the deep postanal space, forming DPAs. Deep postanal abscesses account for approximately 15% to 20% of anorectal abscesses, and they can lead to serious and potentially fatal complications if untreated [4]. Complications include sepsis, fistula formation, and infection spreading to the perineum, scrotum, abdominal area, and/or pelvis [5]. Due to their location, DPAs have a high tendency to form pelvic abscesses and are known to have a higher risk of sepsis [6,7].

The primary goal in managing a DPA is to drain the abscess, eliminate the focus of sepsis, and, if possible, treat the fistula tract. Based on Courtney's theory, open surgical methods, such as the Hanley procedure or modified Hanley procedure, have been widely used to treat deep posterior anal fistulas, as well as techniques such as seton placement or flap advancement. Managing DPAs and related fistulas requires careful evaluation, a strategic approach, and extensive proctological experience. In this case report, we aim to share our treatment details and follow-up on a case of DPA accompanied by osteomyelitis. Such a local sequel of DPA has not yet been reported in the literature.

## Case presentation

A 43-year-old male patient with no comorbidities presented with ongoing coccygeal pain radiating to his back and his thighs. A digital rectal examination revealed a painful swelling in the proximal anal canal. A loose seton had been placed on the left lateral side at another hospital. Contrast-enhanced pelvic MRI was planned with a preliminary diagnosis of anal abscess. Laboratory tests revealed a leukocyte count of 7.8 x 10⁹/L, with neutrophils 5.12 x 10⁹/L. The patient had received oral ciprofloxacin and metronidazole treatment for four weeks at an external center prior to presenting to us. Therefore, we only evaluated the preoperative values upon admission. Since these values were within the normal range due to prior antibiotic use, we did not find it necessary to recheck the laboratory parameters.

**Figure 1:**
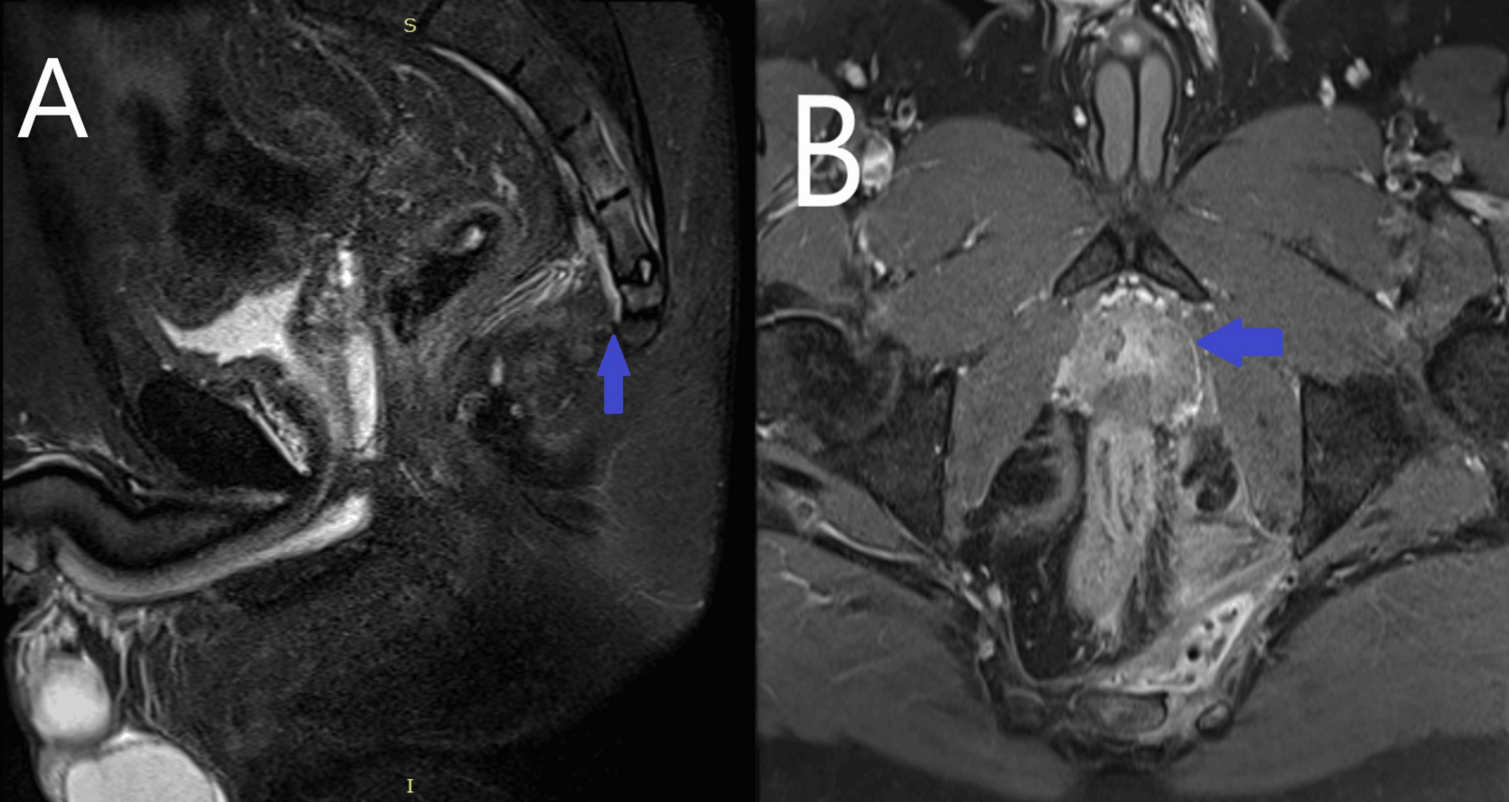
Preoperative MRI images A: On the sagittal short tau inversion recovery (STIR) image, intramedullary hyperintensity consistent with osteomyelitis was observed on the opposite surfaces of the sacral 4-5 vertebrae. Inflammatory hyperintense soft tissue thickening was also noted in the presacral fat tissue. B: On the post-contrast T1 fat-saturated transverse image, an abscess formation containing air with an irregular enhancing wall was seen in the midline-left side of the presacral area, and enhancement consistent with osteomyelitis was noted in the adjacent bone.

The patient had no fever or any septic signs. The MRI images revealed a 5x3 cm lesion consistent with an abscess in the deep anal fossa, with accompanying osteomyelitis at the S4-S5 localization (Figure 1). Although it may not always be necessary, we believe that the use of MRI should be considered in cases of deep postanal abscesses.

**Figure 2:**
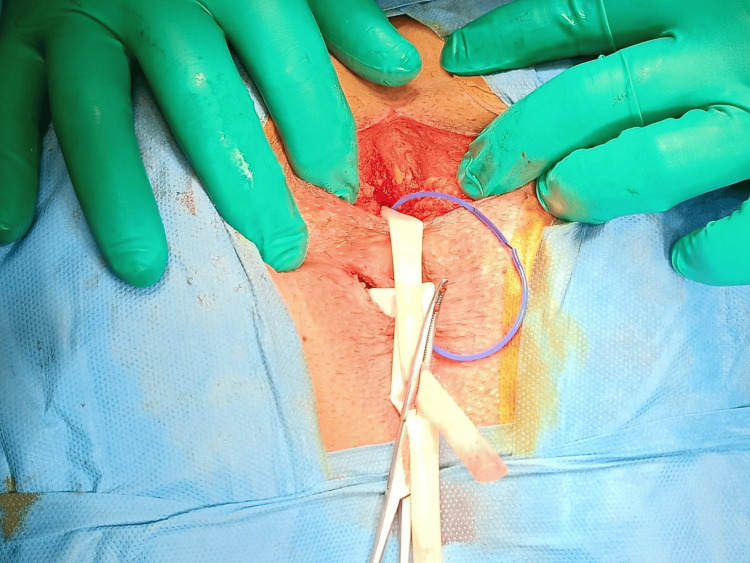
. Intraoperative view The deep postanal space was exposed and drained. A seton was placed between the postanal space and posterior midline intracranial opening, as well as a Penrose drain through the left extension of the deep postanal abscess (DPA).

The previously placed seton was apparently not draining the abscess cavity. The patient was operated on under spinal anesthesia and sedation. In the prone jackknife position, an incision was made on the posterior midline, extending to the coccyx, to reach the posterosuperior part of the external anal sphincter muscle. The anococcygeal ligament was incised, and the abscess cavity was easily exposed. We did not obtain samples from anal abscesses because they are uniformly multi-bacterial with anaerobic components. The patient received only a single dose of IV metronidazole as prophylaxis. After curettage of the abscess cavity, the left lateral deep extension was opened at the skin projection, and a Penrose drain was placed (Figure 2). Subsequently, a seton (SuperSeton®, SuperSeton BV, Amsterdam, The Netherlands) was placed between the deep postanal space and the posterior midline internal opening. The patient was discharged on postoperative day one with the drain and seton in place. Subsequently, two months later, the loose seton was removed, and a transanal advancement flap procedure was performed.

**Figure 3:**
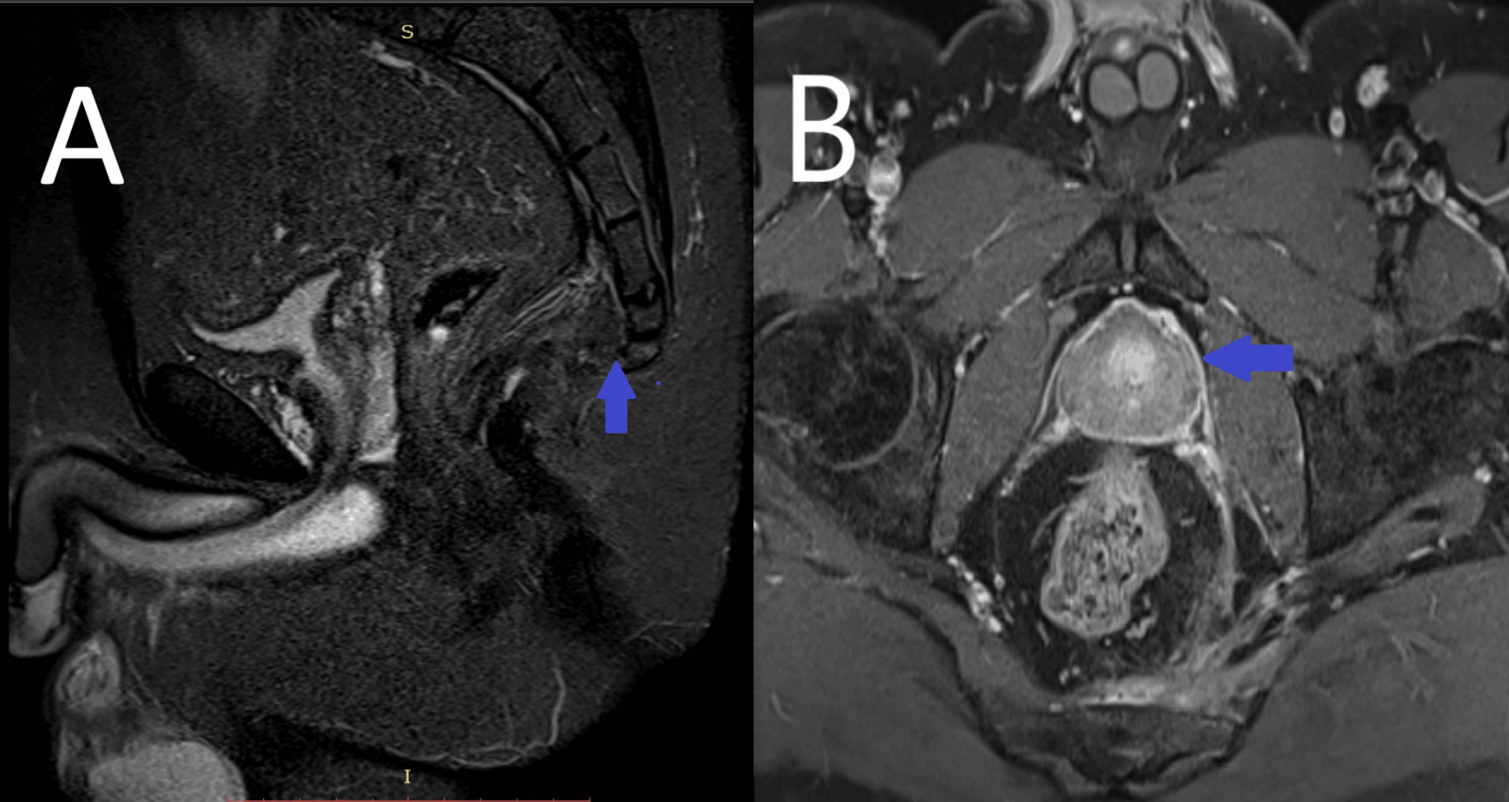
Postoperative MRI images A: After treatment, on the short tau inversion recovery (STIR) sagittal image, the hyperintensity in the vertebrae disappeared, and the inflammatory soft tissue changes in the presacral area significantly decreased. B: On the post-contrast T1 fat-saturated transverse image, pathological enhancement in the vertebrae disappeared, and the abscess pouch was no longer present.

On postoperative day 15, the Penrose drain was removed. The patient declared gradual relief from pain. A follow-up MRI was planned two months postoperatively (Figure 3).

## Discussion

The treatment of DPAs continues to pose challenges for colorectal surgeons in surgical practice. Therefore, careful evaluation and proctological experience are crucial in the treatment of DPA abscesses [2,3].

Sacral osteomyelitis is often caused by bacterial infections, particularly Staphylococcus aureus. The infection can spread from nearby areas or result from direct trauma or surgery. Risk factors include chronic conditions like diabetes, intravenous drug use, or pressure ulcers in immobile patients. Immunosuppressed individuals are also at higher risk. Early diagnosis and treatment are essential for effective management. The treatment of osteomyelitis in the sacral region typically involves antibiotic therapy or surgical drainage and debridement for advanced cases [8].

In our general review of the literature, we did not encounter any cases of sacral osteomyelitis related to DPA. Therefore, we focused on the treatment of DPA in the discussion section. We believe that if the treatment of DPA is successful, the treatment of osteomyelitis will also be successful. Traditional methods, including fistulotomy, seton techniques, and advancement flaps, have varying success rates but often carry a risk of long-term incontinence or require multiple procedures. Ken-Ker Tan and colleagues have proposed a novel intersphincteric approach targeting the infected anal crypt gland. This single-stage technique provides effective drainage with minimal morbidity and avoids extensive incisions. While promising, further prospective studies are needed to confirm its long-term efficacy and impact on continence. In this case, we performed a two-stage surgical approach [9].

In our case, we successfully treated the osteomyelitis by draining the abscess without any further interventions. The patient had undergone antibiotic therapy for several weeks before our treatment. Not surprisingly, this approach did not solve the problem in the presence of an apparent abscess.

## Conclusions

In conclusion, this case describes a very rare case of sacral osteomyelitis secondary to a DPA. Our findings emphasize the importance of surgical drainage in the treatment of sacral osteomyelitis in this framework, and we suggest that the use of antibiotics should be questioned.
